# Case of Congenital Absence of the Vagina, with the Uterus in a Rudimentary State, in Which an Operation Was Performed; with Reflections on the Operations Performed for Absence or Obliteration of the Vagina

**Published:** 1859-07

**Authors:** Daniel Brainard

**Affiliations:** Prof. of Surgery in Rush Medical College


THE CHICAGO

### MEDICAL JOURNAL.

VOL. II. JULY, 1859. No. 7.

### ORIGINAL COMMUNICATIONS.

ARTICLE I.

### CASE OF CONGENITAL ABSENCE OF THE VAGINA, WITH THE UTERUS IN A RUDIMENTARY STATE, IN WHICH AN OPERATION WAS PERFORMED; WITH REFLECTIONS ON THE OPERATIONS PERFORMED FOR ABSENCE OR OBLITERATION OF THE VAGINA.

BY DANIEL BRAINARD, M.D., PROF. OF SURGERY IN RUSH MEDICAL COLLEGE.

The vagina and uterus are subject to every conceivable degree of imperfection from simple narrowing or occlusion from adhesion of the walls to entire absence of one or both these parts.

A systematic classification of these anomalies would range them as follows:

1.—Congenital.

*a.* Imperforation of the vagina from adhesion, or from closure of the hymen.

*b.* Absence of the vagina throughout its whole extent.

*c.* Absence of the vagina and uterus.

*d.* Absence of the uterus with the existence of the vagina.

2.—Accidental.

*a.* Closure of the vagina from adhesions or sloughing (partial).

*b.* The same (complete).

Imperforation of the hymen, or simple adhesion of the sides of the vagina at the external orifice, is an accident of so simple a character, so easily remedied, and of such frequent occurrence, as not to require a lengthened notice. Separating the adhesions by pressure with the fingers or with a blunt instrument, or puncturing the hymen with a bistoury, if necessary, is the treatment demanded. This should be done as soon as the defect is discovered. This precept now generally approved is important for two reasons: because leaving it to obstruct the menstrual flow is dangerous to the health and even to life; and because this closure is often the cause of imperfect development or delay in development of the sexual organs.

This occlusion, and the operation for its relief, were perfectly well known to the earlier writers, and even to the ancients. The fullest account we have of the whole subject is in Morgagni and Aristotle: Hippocrates and Galen were well acquainted with it. It is said that Cornelia, mother of the Gracchi, was affected in this way. It is not, however, to be supposed that the operation of puncture is entirely free from danger. Cases of death from this cause are on record, and will be cited further on, but this seems rather from the effect of distention from retained menses than from the wound itself.

Partial or perfect closure of the vagina or of the os uteri, occurring during pregnancy, so as to obstruct the passage of the child's head, is also an accident so well understood as not to require much comment. All obstetricians agree that in these cases the cicatrices should be incised early, in order to avoid delay and danger of rupture of the uterus. M. Lombard, of Geneva, reported to the Academy of Medicine a case in which the canal of the vagina had been obliterated by injection of sulphuric acid, employed for the purpose of producing abortion. Although incisions were made in this case, they were insufficient, and the patient died after the Cæsarian section had been performed.

I have myself had knowledge of a similar case, in which a piece of whalebone was employed to procure abortion. A firm cicatrix was the result, which, not being recognized by the medical attendant in labor, rupture of the uterus and death resulted.

There is reason to believe that cases of occlusion of the uterus occasionally met with in labor, and of which no explanation is found, are due to attempts to procure abortion.

Accidental obliteration of the vagina.—This accident occurs from pressure of the child's head during labor, improper use of instruments, syphilitic ulcerations, the use of caustic injections, erysipelas following measles or scarlatina, and sometimes without any known cause. I have treated a case in which the inflammation was caused by a disproportion of development of the sexual organs of the male and female. Although the woman in this case was nineteen years of age, the organs were imperfectly developed like those of a girl of fourteen.

Adhesions might, no doubt, be prevented by proper care were danger apprehended, but most frequently it is the impossibility of sexual connection, or the pain occasioned by retention of the menses, which first suggests the necessity of the examination which detects the nature of the difficulty. When the menses return they are accompanied by great pain, which increases at each succeeding period until at length it becomes constant. It is like labor pain, and unless rupture and discharge of the menstrual blood into some internal cavity, or into a passage opening externally should occur, causing death or giving relief, the patient dies, exhausted as in long protracted labor. It is said that the blood sometimes finds its way into the peritoneum, through the Fallopian tube, and thus causes death.

*Treatment.*—The treatment of obliteration of the vagina throughout the whole length is not unattended with difficulty and danger. As the same rules of practice are applicable here as in cases of congenital absence of the vagina with the presence of the uterus, both these cases will be considered together. And in the first place it may, I think, be laid down as a rule that absence or obliteration of the vagina *in unmarried women*, where the menstrual flux does not take place into the uterus, does not require or justify any attempt at operation. It should, whenever its existence is ascertained, be considered an inseparable bar to the contracting matrimonial relations. But when the menstrual fluid is poured into the uterus, symptoms so severe and dangerous are produced as to call in the most urgent manner for an operation. Nor are the reasons which call for the formation of an artificial passage in the married female, in whom no symptoms of the menstrual periods occur, much less urgent. Life is not in danger, but it is without value, as the subjects of the malformation or accident are not only subject to be divorced, but are deprived of the sympathy to which they are entitled, and subjected to the contempt of persons of their own class, who often suppose them to be hermaphrodites.

The operation generally resorted to in these cases consists in making an opening in the natural course of the vagina, and puncturing the uterus in case the os uteri cannot be found. For this purpose a sound should be passed into the bladder and held by an assistant, the index finger of the left hand passed into the rectum. The separation of the membranes of the two organs should be effected as far as possible with the fingers of the right hand, and the scalpel only resorted to when bands are met with which cannot be otherwise overcome. If an incision into the uterus is required, it should be to the extent of three-fourths of a circle, an inch in diameter. This, if kept open for a time with a bougie, has no tendency to close. The bougie or catheter inserted in such cases should be wound below the point with oil silk or india rubber cloth, so as to constitute a sort of pessary an inch or more in diameter, to prevent the closure of the passage. This operation is most conveniently done with the patient in the same position as required for the lateral operation of lithotomy. The probability of being able to establish a communication with the uterus is much greater when that organ is distended by the menstrual fluid than when in a state of vacuity; it is therefore advisable to wait in most cases until the enlargement is ascertained by examination.

The operation, I have said, is not without difficulty and danger. The difficulty results from the liability to lay open the bladder, rectum or peritoneum, or from the impossibility of reaching the os uteri; the danger from the effects of these accidents and from peritonitis, even when no accident has occurred during the operation. The liability to this is so great that many eminent surgeons do not consider the operation as advisable, and recommend puncture through the rectum instead.

Vidal, "Traite de Pathologie Externe," vol. v, p. 618, says: "I have seen this operation performed three times, and each time death has resulted more or less promptly." This he attributes to the passage of air into the uterus, and recommends precautions to prevent it. Mr. Cæsar Hawkins has seen death by peritoneal inflammation result from the puncture of an imperforate hymen, and from puncture of the bladder in another case. DeHaen, in an operation of the kind, opened the bladder, and his patient died. A case is reported in the *Gazette Medicale*, for 1846, p. 57, in which the obstruction seems not to have been perfect, in which a vesico-vaginal fistula is said to have resulted from the operation. Stein and Busch opened the peritoneum, and in consequence their patients died. M. Capuron communicated to the Academy of Medicine a case in which the uterus was punctured from the perineum with a trochar, but permanent relief was not obtained, and the patient died at the end of ten months. Morgagni relates a case in which, after death, the bladder was found to have been opened; but in this instance one of the Fallopian tubes had burst, and discharged its blood into the peritoneum. In a case operated by M. Morrison, an abscess formed in the iliac fossa. A patient of M. Langenbeck died from peritonitis. Dupuytren is said to have frequently seen accidents of an extremely dangerous character result from the operation (Velpeau, Operative Surgery, vol. iii, p. 788, No. 7 edition). In a consultation in M. Amussat's case, Boyer, Marjolin and Magendie advised against any operation. Sir Astley Cooper is said to have cut through two inches of obliterated vagina without finding the os uteri. Mr. Worthington lost his patient, although the occlusion was but half an inch in thickness.

On the other hand, the instances of successful results are so numerous as at the present time to greatly overbalance the unfavorable ones. Among the most noted of these is one reported to the Academy of Sciences in 1832, by M. Amussat, in which an operation was performed on a girl of fifteen years of age for a congenital absence of the vagina. There was retention of the menses. The operation was entirely successful, and the report should be consulted by any one desirous of information on the subject. M. Flamant, of Strasburg, performed a successful operation of the kind. Indeed, successful operations are now so numerous that they cannot all be enumerated. Messrs. Bal, Kluyskens, Gendron, Willhaume, Roux, Nelaton, Mussy (two cases), Mott (said to have operated on a "considerable number"), Harvey de Chegein, DeHaen, Dr. J. M. Warren (four cases), Ventura, Cabaret, Delpech, Desgranges, Renauldin, Keates, Jefferson, Coste, Rossi, Toulmouche, Hayward (two). Reports of these have fallen under my notice, besides many others, in which the occlusion not being complete, they do not come within the scope of this paper.

I have myself met with four cases of accidental obliteration of the vagina. In one already alluded to, the cause was violence from a piece of whalebone used to cause abortion. This resulted in rupture of the uterus and death during labor. Another was caused by the use of instruments in labor, and the entire obliteration of the vagina was accompanied by a vesical fistula. As the menses had not returned, I advised no operation until the uterus should be moderately distended with blood.

The third case was that of Mrs. D., of Wisconsin, a partial report of which was published in the *American Journal of the Medical Sciences*, for October, 1853, p. 365. The exciting cause in this instance seemed to be violence done in sexual connection, an example of which is also furnished in a case of Dr. J. M. Warren. The inflammation was violent; the sloughing extensive; and the closure perfect, both of the entire vagina and of the os uteri. The result of the operation in this was, I think, more favorable than that in any similar case of which I have knowledge. I refer to the greater capacity of the artificial vagina, preserved by keeping for a long time within it a sort of pessary, made with pieces of sponge placed around a piece of wood, and covered with oil silk. Four years after the operation, sexual contact was effected without any perceptible obstruction.

In the fourth case, reported in part with the last, the cause was instrumental labor. The result of the operation was perfectly satisfactory; the menses occurred without difficulty, but the details are not so well known to me.

If any doubt concerning the urgent necessity of an operation in these cases can still be entertained, it will be removed by a glance at a case left to itself.

Dr. J. B. S. Jackson, in 1850, presented to the Boston Society for Medical Improvement a uterus and Fallopian tubes distended with menstrual fluid. The subject, a female of twenty-five years of age, was, in other respects, well formed, but the vagina was wanting. On the discovery of this defect, a consultation of professed surgeons was called, who decided against any operation.

Difficult and dangerous as are these cases of accidental destruction of the vagina, they are not the most perplexing or the most hopeless of the imperfections we are called upon to treat. Cases of absence of the vagina and uterus, or in which these organs exist in a rudimentary state, but in which the breasts and external organs are developed, with all the feminine characteristics, occasionally present themselves under circumstances which appeal strongly to the surgeon for some attempt at relief; and there are some cases on record in which operations have been performed for these malformations, with a certain degree of advantage. Thus Langenbeck, as quoted by Velpeau, succeeded in rendering the vagina permeable in a case in which the uterus was wanting. Dupuytren dissected three inches into the tissue, beyond the extremity of an imperfectly formed vagina, without reaching any uterus. Dr. John Watson, about the year 1844, met with a case in which the uterus was imperfectly developed, and the vagina entirely wanting. There were no signs of menstruation. Nevertheless, Dr. Watson operated by incision, and found the os uteri. The patient recovered, but there was no menstruation, and the opening made contracted to a small size—(*N. Y. Journal of Medicine*, for 1844). It is probable that if a properly shaped pessary had been retained for several months in the passage, or if the patient had been so placed as to have frequent sexual connection, that this contraction of the passage would not have taken place. Dr. E. P. Bennett, of Danbury, Conn., met with a case in which the vagina terminated in a *cul de sac*. Attempts to find the uterus by incision were made without success. Sir Astley Cooper is said to have cut through two inches of cicatrix without being able to find the os uteri. Dr. McFarlane performed a similar operation, but his patient died from peritonitis.

I admit that in case of an unmarried woman, if a surgeon were consulted he should advise against matrimony, and, perhaps, against the performance of any operation. But where a woman has ignorantly entered into the married state and desires an operation, the case assumes a different aspect. The motives which require it are as important to her as if her life were involved; and the question is, whether an artificial vagina, which will be a substitute for a natural one, can be formed without very great danger? To this question an affirmative answer may be given. An artificial vagina is more readily formed in congenital absence than after accidental obliteration. In the latter case, the labia and nymphae are found retracted and diminished in size, from being drawn inward by the contraction of the cicatrix; while in the former, if the sides of the newly formed passage are prevented from coalescing, the mucous tissue of the labia and nymphae, as cicatrization progresses, is drawn inward, expands and developes itself so as to form the walls of the new passage. The mechanism of this has been fully dwelt upon by M. Amussat in the paper already quoted.

Operations for the formation of artificial vaginæ succeed better than *a priori*, we would have reason to expect. The proposition to make an artificial passage in the living tissues in any other part of the body, and to keep it pervious by the presence of a foreign body, would hardly be entertained. But in the case of the vagina, that tendency of the organ to develope itself, which is so marked at puberty, exists for a long time, and is, in my opinion, stimulated into activity by the presence of a foreign body. Certain it is that in two cases the success of the operations which I have performed, with some degree of reluctance, was beyond what I anticipated.

It is not improbable that a simple adhesion of the sides of the orifice of the vagina, at an early period of intra-uterine life, may be the cause of arrest of developement, as we know that adhesion of the prepuce to the glans penis prevents the full developement of the male organs.

It has been noticed too that puncturing the imperforate hymen in young girls gives rise to a temporary sexual feeling of a precocious and somewhat hysterical character.

Without insisting too much upon views which may be regarded as theoretical, I will only premise further that absence of the vagina and uterus is a malformation by no means excessively rare, and therefore important in a practical sense, from its medico-legal as well as from its surgical bearings.

Cases of absence of the uterus, or in which it existed in so imperfect a state as not to be detected by the touch, have been reported by Morgagni (three), Columbus, Fromondus, Haller, Cailliot and Richerand, La Metrie, Baudeloque, Lieutaud, Bosquet, Theden, Rault, Burggrave (who reports two), Bertani, Ramsbottam, Quain, Flamant, Lombard, Boechm, E. P. Bennett, A. Boyd (two), Breschet, Wehr, Seguin, the late S. G. Crawford, of Joliet, Ziehl, Engle, Stein, Madame Chappel, Madame Boivin and Duges, Chew and Murphy.

In the article—"Cas Rares," in the *Dict. Des Sciences Medicales*, by Fournier, published in 1813, it is stated that there existed at that time but one case on record of absence of the uterus. Considering the pains the author had taken to collect together strange occurrences vouched for by doubtful authorities, it is singular that he should have overlooked the four cases related and referred to by Morgagni, in two of which the bodies were dissected, and in the other two the termination of the vagina in *cul de sac* and other appearances, justified him in believing the uterus was absent. It may be stated, however, that in several —perhaps half of the cases above cited—the absence was not proven by dissection, and that the uterus may have existed in a rudimentary state, as was found in a fifth case reported by Morgagni.

In the cases in which the absence of the uterus was not demonstrated by dissection, the conclusion was arrived at by the vagina being either absent or imperfect, terminating in a *cul de sac*, by an examination with a sound in the bladder and the finger in the rectum, by absence of the menses and of all symptoms of their retention.

There was, in nearly all the cases, development of the breasts, of the external organs, sexual desires, and symptoms indicating a tendency to menstruation. These, except the last, depend entirely upon the ovaria, and disappear with their disease or removal.*

CASE.—On the 8th of November, 1856, I was consulted by Mrs. D. on account of some difficulty of the genital organs, which prevented sexual connection. Mrs. D. is twenty-three years of age; has been married one year, but separated from her husband on account of the difficulty for which she consulted me; is well formed, with the breasts and external organs of generation as much developed as is usual, and admits that she has the natural sexual desires. On attempting to pass the finger into the vagina, it was arrested immediately behind the orifice. On ocular examination, there is perceived a small *cul de sac*, the membrane on the surface of which lies in folds. On passing a sound into the bladder and the finger into the rectum, the septum between these organs was found thick. On careful and repeated attempts, however, a probe was passed from the orifice of the vagina, and between the bladder and rectum, a distance of two and a half inches, without using violence. Beyond this, and as far as the finger could reach, a small body was felt which seemed to be the uterus, of the size of that of a child of five years of age, and bent with the inflexion backward.

The orifice of the urethra bore marks of violence, which, I afterwards learned, had been produced by the forcible introduction of the finger by the patient herself; and I also ascertained that in her anxiety to restore the natural passage, she had thrust into the vagina, or its natural situation, pieces of whalebone, some of which had apparently entered the rectum, as a discharge of blood from that organ followed. I do not, however, think that the canal which the probe followed was formed in this manner, as the wound was entirely healed while this canal persisted.

This woman and her mother denied having had any knowledge or suspicion of the existence of this obstruction before marriage. She had never had any attack of inflammation of the organs. At times she had the usual symptoms attendant on menstruation,

---

* See Bowen and Dayes, Maladies de Uterus, Potts' Surgery, etc.

but not with regularity, although she had sometimes had discharges of blood from the rectum.

By repeated and careful examinations per rectum and on the surface of the abdomen, no tumor of any kind could be detected. I therefore came to the conclusion that it was a case of absence of the uterus and vagina, or one in which these organs existed in a state of very imperfect development.

The important question to the patient was the possibility of performing any operation with advantage. At first I advised against any attempt of the kind, but after consulting several eminent colleagues, and particularly Dr. John Evans, formerly professor of obstetrics in the medical college at Chicago, I was iuduced to attempt a dilatation of the small canal already spoken of. This was done at first with a grooved director, upon which a metallic director was passed. The patient was unable to bear the pain produced by these instruments if of considerable size, and this plan was abandoned.

Bearing in mind the favorable result of my operations for obliterated vagina, and having in this case a guide which obviated the danger of wounding the bladder or the rectum, I determined to enlarge the canal by incision. This was done, Dec. 27, 1856, with the assistance of Drs. Evans, Isham and Nutt, as follows:

The incisions were made laterally, and the tissues separated as much as possible by the finger. The small body which I took for an embryo uterus was reached. It had, however, no cavity, and the operation was finished by passing into the opening a small rod surrounded by turns of india rubber tissue until it was one inch in diameter.

The operation was not extremely severe; the patient being under the influence of chloroform suffered no pain. The hemorrhage was not great. A full dose of morphine was administered.

Considerable reaction occurred during the first week, the bladder particularly suffering from inflammation. Under appropriate treatment these symptoms subsided. The most painful part of the treatment was putting in and taking out the pessary, which was therefore done at as long intervals as possible.

At the end of about a month the patient returned home, with the direction to wear the pessary constantly for some months, and then to commence leaving it out a few hours at a time.

At the end of fourteen months—viz.: in March, 1858—the patient returned. She had been in good health, able to do the work of the house most of the time. She had continued to wear the pessary most of the time, taking it out and replacing it herself as occasion required.

At this time she was examined by Dr. Byford. We found the artificial vagina sufficiently long to admit the index finger its whole length, and broad enough to allow of free lateral movement. The surface was soft and flexible, and did not appear to secrete pus. The body which I have taken for a uterus was not perceived by this examination, and no examination *per rectum* was made. Dr. Byford and myself were of the opinion that copulation might be perfectly effected, and that even a skillful surgeon, in making an examination at this time, would not suspect the degree of imperfection which had formerly existed.

In May, 1859, two years and four months after the operation, the patient was examined by several physicians in Wisconsin, who report her condition the same as when she was last examined by Dr. Byford and myself.

In so far as a single case may be taken as a guide, this seems to indicate the propriety of an operation when the vagina and uterus are both in a rudimentary state, a condition hitherto generally believed to be beyond the resources of art.

